# Educating for diversity, equity, and inclusion: A review of commonly used educational approaches

**DOI:** 10.1017/cts.2021.834

**Published:** 2021-08-09

**Authors:** Leonor Corsino, Anthony T. Fuller

**Affiliations:** 1 Department of Medicine, Division of Endocrinology, Metabolism, and Nutrition, Duke School of Medicine, Durham, North Carolina, USA; 2 Duke Clinical and Translational Science Institute, Community-Engaged Research Initiative Core, Duke School of Medicine, Durham, North Carolina, USA; 3 Department of Neurosurgery, Division of Global Neurosurgery and Neurology, Duke University School of Medicine, Durham, North Carolina, USA; 4 Duke Global Health Institute, Duke University, Durham, North Carolina, USA; 5 Duke Clinical and Translational Science Institute, Center for Pathway Programs, Duke School of Medicine, Durham, North Carolina, USA

**Keywords:** Diversity, equity, inclusion, education, bias training

## Abstract

Diversity, equity, and inclusion (DEI) are fundamentally important concepts for advancing clinical and translational science (CTS) education. CTS education spans a wide range of disciplines from cell biology to clinical and community/population research. This large scope both in terms of intellectual areas and target groups requires an understanding of existing educational approaches for DEI as we translate DEI from mere concepts into equitable actions within CTS education. In this  review, we provide the readers with the most common DEI educational approaches, including cultural humility, bias training, and improving mentoring to diversify the workforce. DEI educational materials can achieve maximal success and long-term impact when implemented as institutional-wide interventions, and the materials are not seen as an isolated or independent curriculum. Approaches, strategies, and programs to achieve this are many. However, many questions remain unanswered about what the best approach, strategies, and programs are to be implemented in institutional-wide education that will be embedded in CTS education.

## Background

Diversity, equity, and inclusion (DEI) are fundamentally important concepts for advancing clinical and translational science (CTS) education. In recent years, increased societal consciousness has led to the precipitous rise in the usage of DEI in everyday vernacular. Often, when concepts become ubiquitous, their meanings morph such that individuals may have completely different ideas of the concept’s definition, or they become “code” that can lead some audiences to opt-out.

For the purpose of this review, we use diversity and inclusion as concepts in alignment with the National Institute of Health (NIH) definitions, given the NIH’s position as a major funder of CTSs. Diversity is defined by the NIH as “the range of human differences, including but not limited to race, ethnicity, gender, sexual orientation, age, social class, physical ability or attributes, religious or ethical value system, national origin, and political beliefs” [1]. While inclusion is defined as “involvement and empowerment, where the inherent worth and dignity of all people is recognized” [1]. For equity, we define this concept as “the state, quality, or ideal of being just, impartial, and fair” [2].

DEI work in CTS education requires an intentional focus on those already practicing in CTS as well as a focus on the training and education for the next generation of practitioners and investigators. CTS is uniquely positioned to reinforce inequities or entirely reshape and reduce inequities; therefore, the additional focus on trainees allows influence not only on the scientific questions that will be asked but also the scientific environment that they will inhabit. CTS education also spans a wide range of disciplines from cell biology to clinical and community/population research [3]. This large scope both in terms of intellectual areas and target groups requires an understanding of existing educational approaches for DEI as we translate DEI from mere concepts into equitable actions within CTS education.

CTS education is positioned within an academic environment that will either support or stifle DEI efforts. Environmental factors contributing to the support or stifling of DEI efforts include the makeup of the institution’s faculty, staff, leaders, and students alongside the institution’s evaluation procedures and policies. These environmental factors are distinct from educational materials created specifically to increase an institution’s members’ understanding of DEI. It is entirely plausible that an institution can have high-quality, innovative, and well-intended educational DEI materials within an environment that hinders its impact on its CTS educational community. The converse is also true. This reality is why we believe that within the CTS education community, despite our paper’s focus on DEI educational approaches, we must not only focus solely on the development and implementation of high-quality educational DEI materials, but also invest in cultivating just, equitable, and supportive learning environments.

While preparing for writing this review, we approached the intersection of DEI within CTS education with the broadest scope possible and then began narrowing. Our initial search terms brought up thousands of articles that spanned the spectrum from articles about educational environments to DEI materials. We choose to focus our paper on DEI educational materials and approaches, which narrowed the articles to a more manageable range. Each article in our search was examined to develop a list of the most common DEI educational approaches, and then we dove deeper into each approach to highlight the most salient features for the CTS educational community.

## Educational Approaches and Programs

Several approaches have been pursued for educating DEI. In this section, we will provide a summary of some of the existing educational approaches and programs created and developed to enhance cultural humility, ameliorate bias, and improve mentoring to diversify the workforce in science. We recognize that due to the increasing body of literature in this significance and evolving area of research and education, it is impossible to be fully inclusive of all the work that has been done and is ongoing.

### Cultural Humility

Cultural humility is defined as a lifelong process of self-reflection and self-critique whereby the individual not only learns about another’s culture, but one starts with an examination of her/his own beliefs and cultural identities [4]. Cultural humility training, usually referred to as cross-cultural training and education, is commonly offered to an array of professionals. The main goal of this training is to enhance cross-cultural interactions and increase personal awareness of one’s values and beliefs to increase the understanding and acceptance of others [5,6].

Although training in cultural humility is not new and has been implemented for decades, the approaches to implementation and its inclusion in research training are relatively new [4]. Traditionally, training to increase cultural humility utilizes workshops as its main pedagogical method [6]. These workshops are usually didactic, delivered for several hours or 1–2 days. This can lead many to perceive them as superficial. Further, those who are compelled to attend may find them divisive and uncomfortable [6,7].

Cultural immersion, based on the principle that immersion in another’s culture, practices, and language is an effective means of learning about oneself “in” another culture, has been utilized as another method to teach cultural humility [8]. Cultural immersion focuses on (1) increasing students’ capacity for empathy by exposing them to a different worldview, (2) developing critical self-reflection/self-awareness, (3) experiencing traditional cultural practices, and (4) exploring traditional and contemporary values and beliefs, focusing on the culture’s strengths [8]. Several studies have documented the impact and benefits of cultural immersion as a method to teach cultural humility [9]. Similarities among the studies include short-term immersion into a culture different than own, reflective journaling, daily writing, and debriefings [10].

A recently published systematic review looking into cultural immersion educational programs for healthcare professionals reports a total of 9 studies with a total of 94 participants with experiences in 14 culturally diverse environments. The interventions and assessments utilized by each program include didactic lecture, study abroad experience, semi-structured interviews, focus groups, journaling, and reflective papers. The authors concluded that participants in immersion programs demonstrated growth in the cognitive, affective, perpetual, cultural dissonance, and skills/engagement domains. The paper concluded that cultural immersion experiences can produce a positive multidomain effect in its participants.[10].

New approaches to delivering cultural humility training have been proposed. A group of investigators from the Rush Institute for Healthy Aging proposed the QIAN (Humbleness) curriculum: the importance of self-questioning and critique, bi-directional cultural immersion, mutually active listening, and the flexibility of negotiation curriculum. The QIAN curriculum is based on Chinese philosophy and is inspired by ancient Chinese thinkers. The investigators proposed a model that incorporates the following: (1) Question asking: questions regarding our own assumptions about the world, where the assumptions come from, constant self-questioning and self-critique; (2) Immersion: immersion that goes beyond exposure to other cultures; (3) Active listening: active listening with the body (gestures and body languages), mind (stories and narratives), and soul (feelings and emotions); and (4) Negotiation: willingness to negotiate mutually acceptable alternatives carries equal weight as learning each other’s preference. [7]

Another approach proposed to deploy cultural humility training includes an art-based curriculum. Art-based training for cultural humility has been proposed as an innovative and creative way of training health professionals. Art-based interventions that highlight self-reflecting artmaking facilitated insight, understanding, awareness, and competency [11].

Simulation is another method proposed for increasing cultural humility. Simulation for developing cultural humility has been utilized as a new pedagogical approach in nursing [12–14]. A review article published in 2017 looking at Cultural Competency and Cultural Humility in Simulation-Based Education identified a total of 16 studies. Within the 16 studies included in the review, a total of four themes emerged: (1) cultural sensitivity and cultural competence, (2) insight and understanding, (3) communication, and (4) confidence and comfort. However, the methods varied widely within these studies. At the end, the authors concluded that no one study existed at the time that describes the use of simulation to teach cultural humility [15]. Since the publication of this article, several others have shown the utility and the need of simulation as a new and innovative method to teach cultural humility [16,17].

### Bias Training

Bias, conscious, or unconscious has been cited as a major contributing factor in health and health care disparities and underrepresentation of historically minority groups in science and academia [18]. The term “implicit bias” or “unconscious bias” gained significant attention and has been the subject of many publications. The “unconscious bias hypothesis” which is widely quoted in social psychology research, portends that bias can occur without recognition [19]. Bias is usually referred to as both stereotypes and prejudices and as “the negative evaluation of one group and its members relative to another” [20]. While studies have documented bias in health care delivery [21], additional research has shown the impact of unconscious bias in research, admissions, hiring policies, and underrepresented minorities (URMs) progression in academia [22–24].

To educate for DEI, it is necessary to address the significant impact that bias plays in our day-to-day lives as researchers, health care providers, educators, and leaders. The recognition of the impact of bias in all aspects of academic medicine is the main force behind the increasing number of materials and approaches developed and implemented to increase awareness of bias and its impact. Although it is not possible to eliminate our own unconscious bias, it is potentially possible to ameliorate its impact on our decisions while treating patients, conducting research, interviewing, and leading [25].

Numerous programs, educational materials, and approaches have been developed to address bias. It is challenging to provide a complete summary of the existing data and publications pertaining to unconscious bias due to the exponential increase in the number of publications within the last decade. However, for the purpose of this review, we will provide the readers with the most common approaches utilized and proposed to increase awareness, knowledge, and skills development to address the impact of bias in all aspects of academia including CTS education.

### Awareness, Knowledge, and Skills

To address biases, we need to become aware that they exist and their impact on behavior. Approaches to increased awareness are currently being implemented. One highly utilized tool is the Implicit Association Test (IAT). The IAT is currently the only available objective measurement of unconscious bias. The IAT measures the differential association of two target concepts with attributes. IAT, developed in 1998 by Banjani and Greenland [26], has been extensively utilized by many studies addressing unconscious bias [27]. Although the IAT is widely utilized and there is research proving its validity [28], there is some controversy regarding it’s utility [29]. One of the main critiques of the test is to what extent awareness predicts behavior [30]. Despite the limitations of the test, its utility to increase awareness and its free availability makes it a valuable tool for bias awareness.

Research and publications reporting curriculum and programs developed to address the issue of racial bias in academic medicine are vast. The research ranges from programs targeting medical students [31,32], residents [33], faculty [34], and search committees [35]. Overall, commonalities within these programs and educational materials comprise the use of workshops, multimedia presentations, small group discussions, interactive audience polling, self-reflection, and clinical vignettes or case studies.

Educational materials focused on interventions to acquire skills to reduce the impact of bias are less commonly reported. However, some information exists regarding strategies to prevent implicit bias. Four strategies that show potential for reducing implicit bias include: (1) pursuing egalitarian goals by learning to associate minority groups with goals that promote fairness and equity, this potentially helps cutting the stereotype off even before they appeared; (2) identifying common identities by shifting the attention from differences and focus more on common interests and activities; (3) counter-stereotyping by focusing on the individual unique attributes and behaviors; and (4) perspective-taking by taking the perspective of the minority group [36].

### Improving Mentoring to Impact Clinical Translational Science Education

CTS education will not be successfully achieved without deliberate attention to improve mentoring to diversify the workforce. Diversifying the workforce has been recognized as an important and necessary priority to further scientific discoveries, eliminate health disparities, improve minority health, and achieve patient-centered outcomes [37]. Robust mentorship has been cited as a way to enhance workforce diversity in health sciences and research [38]. Research has shown that trainees from URM groups receive less mentoring than their White peers [39]. Further, improving mentoring to increase DEI in research has been identified as a priority by the NIH [39]. The NIH directly addressed the science of diversity, citing the racial, ethnic, gender, and economic balance of the US biomedical research workforce as limiting the promise of building knowledge and improving the nation’s health [40]. To that end, the National Research Mentoring Network (NRMN) a nationwide consortium of biomedical professionals and collaborating institutions sponsored by the NIH works to provide all trainees across scientific disciplines with evidence-based mentorship and professional development programming that emphasizes the benefits and challenges of diversity, inclusivity, and culture within mentoring relationships and, more broadly, the research workforce. The goal of the NRMN is to increase the diversity of biomedical research by enhancing the mentorship and career development of individuals from diverse backgrounds, communities, and cultures [41].

The evidence-based curriculum, activities, and training resources available via the NRMN are grounded in a robust conceptual model, authentically address bias, stereotype threat, and cultural ignorance, focus on the formal preparation of both mentors and mentees, builds upon process-based, community-building approaches to mentor and mentee training, and include established multimodal training formats and proven train-the-trainer efforts that allow for rapid scale-up and sustainability.

Considering the extensive efforts by the NRMN in the development of a publicly available curriculum to train mentors and mentees to improve mentoring practice that will lead to DEI in research, we encourage others to explore and engage in activities to deploy this training widely. We recognized that there are potential limitations experienced by some academic institutions to fully deploy the curriculum, such as lack of time, financial support, and other resources including trained facilitators. However, it is challenging to educate for DEI when diversity in the scientific workforce is not achieved.

The NRMN curriculum has been adapted and implemented successfully by Clinical and Translational Science Awards (CTSAs) around the country. Through the Institute for Clinical and Translational Research (ICTR), the *Entering Mentoring* training materials were adapted for use with CTSA mentors. In a randomized controlled trial, the entering mentoring materials were implemented at 16 CTSA institutions across the country [42]. In this study, a total of 283 mentor–mentee pairs were recruited. Mentors were randomized to the 8-hour training group or to the control group. The curriculum is implemented in a small group of mentors that engage in discussions based on case studies and activities. The curriculum was deployed by two facilitators and in four 2 hours sessions. The curriculum focuses on six core competencies: (1) maintaining effective communication, (2) aligning expectations, (3) assessing understanding, (4) addressing diversity (5) fostering independence, and (6) promoting professional development. Evaluation of the curriculum demonstrated improvement in mentors’ skills important for successful mentoring such as communication and evaluation skills [43].

## Implementation, Dissemination, and Evaluation

Increased attention and focus on DEI has led to the development of a wide array of educational materials with varying levels of quality and distinct pedagogical approaches. Sifting through the options to select the best and most impactful approaches requires the same attention to detail and scientific rigor as any other topic in CTS. Practically, this means that deliberate attention is given to the selection of educational materials, to the choice of the faculty, staff, and students who will administer and receive the educational materials, and to the environment in which the materials are being implemented.

DEI educational materials can achieve maximal success and long-term impact when implemented as institutional-wide interventions, and the materials are not seen as an isolated or independent curriculum. DEI education must be viewed as integral and intertwined with the successful mastery of every topic and aspect within CTS. Programs and institutions across the country are at different stages in the process of fully integrating DEI into their curriculum. Most have communicated acknowledgment of DEI’s importance by placing it within their mission statements. Undoubtedly, this is an important step towards full-scale systemic changes in the structures, environment, and educational materials.

As programs and institutions begin their journeys in DEI development and integration, dissemination becomes imperative. Dissemination serves a tripartite purpose by providing a channel for iteration, refinement, and sharing of best practices. The Association of American Medical Colleges’ (AMMC) MedEdPORTAL Diversity, Inclusion, and Health Equity Collection is a good example of a dissemination platform (Table 1). Through this and other mechanisms, work being done at a single program or institution can contribute to the growing body of work in this space. Collective knowledge development through dissemination is a key lever for success as programs and institutions grapple with the daunting task of dismantling racism, sexism, ageism, ableism, and a multitude of other isms.

**Table 1.** Curriculum and educational approaches and materials resources

Topic	Resources
Anti-racism collection	https://www.mededportal.org/anti-racism https://www.diversityprogramconsortium.org/pages/anti-racism_resources
Diversity, inclusion, and health equity collection	https://www.mededportal.org/diversity-inclusion-and-health-equity
Mentoring training resources	https://nrmnet.net/#undergradPopup https://nrmnet.net/senior-faculty/ https://cimerproject.org/online-resources/ https://cimerproject.org/culturally-aware-mentoring-resources-2/
Diversity research collection	https://diversity.nih.gov/find-read-learn/diversity-research-articles

Rigorous evaluation of DEI educational interventions is an additional lever for success. There is a desperate need to try to get this “right,” which means there must be a way for CTS educators to know which DEI educational materials are better and what impact are to be expected. Checklists, audits, toolkits, and evaluation surveys have already been created [44].

## Unmet Needs and Barriers

Institutional and program willingness, adequately trained and resourced staff, and receptive students are only part of the complex puzzle of educating for DEI in CTS. Unmet needs are embedded and widespread within each of these areas. Most institutions and programs have a general willingness to engage in DEI work and are faced with resistance [45].

Across the board exists the need to see the value and then to invest the time, funding, and development of qualified instructors. Until recently, DEI work has been an afterthought or has garnered increased attention due to tragedy and exposure of inequities.

## Putting DEI into Practice

Academic institutions and CTSAs within these institutions recognize the value of DEI in the advancement of sciences. As such, implementing approaches to further educate stakeholders for DEI are important. Our simple conceptual framework focused on two distinct ideas: the creation of a conducive environment and the creation and implementation of educational materials and curriculum. The framework highlights the importance of the environment when it comes to fostering DEI. Without a supportive and conducive environment, advancement to ameliorate racism and bias in research and academic institutions is close to impossible.

Although, in this study, we focused mostly on describing some of the most used approaches to educate for DEI as we cannot overemphasize the impact of the environment. To implement training in cultural humility, bias training, and mentoring training, it is critical to have an environment that supports these initiatives. For example, the testing and implementation of mentoring training at several CTSAs around the country were possible with the support from NIH funding and buy-in by CTSAs leadership.

Similarly, training in bias and cultural humility requires dedicated effort to hire, train, develop, and implement new and existing materials. To that end, the creation of diversity and inclusion offices, centers for equity, and institutes dedicated to these efforts are important and, as such, should be fully supported and resourced. Also, the efforts to educate for DEI are no longer isolated and are becoming more and more critical components of research, training, and education. However, more is still needed. For example, validated measurements to assess the short and long-term impact of bias training. In the meantime, to what extent training that aims to change very rooted bias has an impact on research remains unknown. Finally, there is a need to continue the conversation, the creation, implementation, research, and innovation in DEI education.

## Conclusion

Educating for DEI and dismantling racism in research and academic institutions is a national priority. Approaches, strategies, and programs to achieve this are many. However, many questions remain unanswered pertaining to what the best approach, strategies, and programs are to implement institutional-wide education that will be embedded in CTS education. Further, as we continue to explore, test, and implement these approaches, strategies, and programs, other questions remain regarding the best assessments to determine their impact.
